# Correlation of Hepatitis C Antibody Levels in Gingival Crevicular Fluid and Saliva of Hepatitis C Seropositive Hemodialysis Patients

**DOI:** 10.1155/2009/247121

**Published:** 2009-09-10

**Authors:** Gökhan Açıkgöz, Murat İnanç Cengiz, İlker Keskiner, Şereften Açıkgöz, Murat Can, Aydan Açıkgöz

**Affiliations:** ^1^Department of Periodontology, Faculty of Dentistry, Ondokuz Mayis University, Samsun, Turkey; ^2^Department of Biochemistry, Faculty of Medicine, Karaelmas University, Zonguldak, Turkey; ^3^Department of Oral Diagnosis and Radiology, Faculty of Dentistry, Ondokuz Mayis University, Samsun, Turkey

## Abstract

Search for hepatitis C virus (HCV) in body fluids other than blood is important when assessing possible nonparenteral routes of viral transmission. However, the role of oral fluids in HCV transmission remains controversial. Our aim was to compare the prevalence of HCV antibody (HCV Ab) levels in saliva, and gingival crevicular fluid (GCF) of HCV seropositive hemodialysis patients. Serum, saliva and GCF samples were collected from thirty-nine patients. Samples were analyzed for HCV Ab using the Ortho HCV 3.0 SAVe enzyme-linked immunosorbent assay (ELISA). HCH Ab levels in saliva and GCF of all HCV-seropositive patients were statistically compared. Reported here are the results of the study designed to determine the correlation between HCV-RNA positivity in serum and the detection of antibodies in GCF and saliva. One hundred percent (100%) of the 39 patients have antibodies to HCV in their serum, 15.4% have antibodies to HCV in GCF, and saliva found out. HCV Ab seropositivity in GCF and saliva was significantly correlated (kappa = 0.462; *P* < .001). This study supports the concept that GCF may be a significant source of HCV in saliva.

## 1. Introduction

Hepatitis C virus (HCV) infection is associated with a poor prognosis for survival among dialysis patients [1]. Reports on hemodialyzed patients from various countries screened by serological assays and/or RT-PCR have shown a prevalence of 12–29% [2]. The diagnosis of infection with HCV is based on the detection of HCV-specific antibodies (HCV Abs) in serum or plasma. However, during the past decade, several authors have described the suitability of alternative samples, such as saliva and Gingival Crevicular Fluid (GCF). The detection rates of HCV RNA in various studies of saliva have ranged from 0% to 100% [3–9]. The mechanism of appearence of HCV in saliva is unclear. Although HCV is considered essentially hepatotropic, some studies have suggested that viral replication occurs also in peripheral Blood Mononuclear Cell (BMC) and in the submaxillary glands [10–12]. However, possible source of HCV in saliva may include serum exudate, that is, the GCF and the migration of HCV-containing mononuclear cell from periodontal inflammation at the dentogingival interface into the salivary pool. There are limited studies wich have qualitatively identified HCV in GCF; HCV RNA was detected in 59% and 85% of GCF specimens from HCV patients [13, 14]. Since the efficiency of HCV transmission is likely related to its viral load, it is important to identify quantitatively the viral RNA levels within the body fluids in order to properly evaluate possible nonparenteral routes of HCV infection. However, in the general population, periodontal disease is common and may influence the increase of mononuclear cell excretion in GCF [15].

Several oral fluid collection methods have been developed; saliva testing had a similar or better specificity than the serum method [16]. Currently, routine diagnosis of HCV is based on detecting antibodies (HCV Abs) in serum by ELISA [17]. Saliva is easy to obtain, especially in outdoor setting and in children. Thus, detecting infections using saliva samples may be of significant clinical, economical, and epidemiological importance [16]. However, as the concentration of Ig G in oral fluid specimens collected by any device may still be lower than that in serum, serological tests originally developed for serum or plasma have been modified to enhance their sensitivity. These modifications include increasing the sample volume, lowering the sample dilution, increasing the sample and conjugate incubation times, and optimizing the cutoff. In previous studies [18, 19], sensitivity and specificities for HCV Ab detection were 94.4–99.1% and 98.2–99.1%, respectively, depending on the oral fluid collection and the HCV antibody screening assay used.

Although there was not a statistically significant correlation between the serum viral load and HCV level in saliva or GCF, patients with low serum HCV loads were less likely to have detectable HCV in their saliva. These findings have important implications for medical personnel and suggest that epidemiological studies designed to understand the significance of the oral route to transmission of HCV are warranted [14].

The presence of HCV in the saliva has been shown in hemodialysis patients [20, 21]. Hence, the risk of contamination should be considered. Therefore, a potential source of HCV RNA within saliva includes GCF, which might contain HCV-infected BMC in setting of periodontal inflammation [13, 14]. Also, it has been shown that rduction in lacrimal and salivary secretion is frequent in dialysis patients [22]. To our knowledge, there is no any study identifying HCVAb in GCF in the patients with hemodialysis.

Thus, we examined the prevalence of antibody to HCV in saliva and GCF of HCV seropositive hemodialysis patients using ELISA assay in oral fluid specimen as a possible alternative to serum [16, 23].

## 2. Materials and Methods

Thirty-nine dialysis patients (22 male and 17 female) participated in the study. All patients had been admitted to medical and dental school clinics. Blood samples from all patients, on hemodialysis at Ondokuz Mayis University, Hemodialysis Center in Turkey, were taken on a monthly basis for the detection of anti-HCV Ab, and in addition twice per year for the detection of the presence of HCV-RNA. Anti-HCV antibodies (HCV Abs) were analyzed using Ortho HCV 3.0 SAVe ELISA (Ortho-Clinical Diagnostics, Inc. Raritan, NJ). An assay commercialized for serum testing has recently been adapted for oral fluid testing, obtaining a sensitivity and a specificity of 91.7% and 99.2%, respectively [23]. Testing for HCV antibody (HCV Ab) in GCF and saliva was carried out in the patients whose sera were determined to be positive for HCV-RNA and HCV Ab. Before sample donation, all patients provided informed consent and the protocols were approved by the Institutional Review Board. The patients with a history of alcoholism or exposure to hepatotoxic drugs and presence of hepatitis B surface antigen, antinuclear antibody and antibodies to HIV-1 and HIV-2 and the patients having HCV-RNA positive but not HCV Ab in serum were excluded from the study. At the time of the study, none of the patients was receiving any specific antiviral treatment. Liver function tests in all the patients were within normal limits. The patients were advised not to eat or practice oral hygiene for 2 hours prior to the procedure.

### 2.1. Collecting of GCF and Saliva

Blood and saliva samples were obtained from each patient attending the unit of Nephrology prior to hemodialysis. Each participant donated two-three blood and saliva samples on different days. Blood samples were collected in sterile tubes, and saliva samples were obtained by asking the participant to spit into a sterile plastic cup. GCF specimens were collected by first drying the gingival surface with sterile cotton, after which the area was isolated in order to prevent contamination with saliva. A paper strip (2 by 5 mm) was then subgingivally inserted for 30 seconds to collect specimen. Saliva and GCF samples were examined for the presence of red blood cells by Orthotolidine method expressing positive reactions with dilutions of 1 : 100 000. [24], and processed immediately. In case of visible blood contamination, the samples were rejected and another was taken from another site. After 30 seconds, the paper points with absorbed fluid were placed into plastic vials containing 500 mL of Phosphate Buffered Saline (PBS) with 0.05% Tween 20 and immediately put on the rocker for one hour at room temperature for the elution of gingival fluid. None of the samples contained blood. Blood, saliva, and GCF samples were centrifuged immediately at 3000 rpm, for 15 minutes at 4°C. All the samples were collected simultaneously and were stored at –80°C before use.

### 2.2. Laboratory Methods

All serum, GCF, and saliva samples were tested using Ortho HCV 3.0 SAVe ELISA, which detects anti-HCV antibodies. Saliva and GCF samples with optical-density values falling between 0.020 and 0.060 were considered indeterminate (Gray Zone) and were retested before final interpretation. Final values equal to or greater than 0.035 were considered positive and values lower than 0.035 negative. Serum HCV-RNA was tested by using the Cobas Amlicor HCV 2.0 Assay (Roche Diagnostic System, Basel, Switzerland), according to the manufacturer's instructions to assess active viral replication. The lower detection limit of this assay was 50 IU/mL.

### 2.3. Statistical Analysis

Descriptive statistics were performed using the Chi-square test and its modification, McNemar's Chi-square test, which was used for the paired proportions. Statistical analysis was performed using the SPSS 11.0 program, *P* values <.05 were considered statistically significant.

## 3. Results

All the patients were HCV-RNA and HCV-Ab positivity in their serum were studied. In 74.4% of the HCV seropositive subjects studied HCV-Ab was found to be negative in GCF with 10.3% of the subjects in the gray zone and the remaining 15.4% exhibiting positve results in GCF. In saliva, 79.5% had negative, 7.7% had gray zone, and 12.8% had positive results. In both GCF and saliva 66.7% of the subjects were found to be negative. In 5.1% of the subjects HCV- Ab was found to be positve. 10.3% of the subjects exhibited positve results in GCF as 7.7% of the subjects had positve results in their saliva. A total of 5.1% of the patients had both HCV-Ab positive GCF and saliva, while 66.7% both fluids were HCV-Ab negative (Table 1). The correlation coefficiency (kappa) between GCF and saliva HCV levels was 0.462; (*P* < .001). Mc Nemar's chi-squared test revealed no statistically significant difference in HCV immunopositivity between saliva and GCF (*P* > .05).

## 4. Discussion

There is considerable amount of evidence to suggest a nasocomial transmission of HCV within the hemodialysis units, although the exact modes of transmission are not fully clarified. A nasocomial transmission of HCV infection in dialysis units has recently been documented using molecular techniques [25]. Transmission of HCV through parenteral exposure has been well documented. However, in 30–40% of HCV positive patients do not have a history of parenteral exposure [26]. Although several studies have investigated the presence of HCV in saliva, the levels reported have varied considerably from 0 to 100% [3–9, 13, 14, 27–33]. The detection of HCV-RNA is indicative of the presence of HCV particles in the saliva, thus establishing saliva as a potential carrier of infection, confirming the possibility of a nonparenteral route of transmission for HCV. Indeed, the potential to transmit HCV by biting has been reported in a chimpanzee model and in humans [31–33]. In contrast to some studies, HCV-RNA was extracted from the whole saliva [6, 34]. The presence of HCV-RNA in some cells has been described, although the presence of HCV-RNA in these cells did not correlate with the presence of HCV-RNA in the serum [11].

The role of oral fluids in HCV transmission remains controversial. Although the presence of HCV-RNA in saliva has been reported by several research groups, only limited studies have attempted to identify HCV-RNA in saliva, in which patients coinfected with HCV and HIV were examined using a branched DNA assay [35]. Few studies have investigated the occurence of human viruses in GCF [13, 14, 36, 37]. Recently, the analysis of GCF and saliva for the concentration of HCV-RNA revealed a higher detection rate and greater levels of RNA in GCF versus saliva [13, 14]. These data support the concept that GCF may be a significant source of hepatitis virus in saliva.

Here, we observed HCV Ab more commonly in the GCF than the saliva of HCV-seropositive hemodialysis patients (Table 1). Our findings are in accordance with those of Matičič et al. [13] and Suzuki et al. [14]. Forthermore, we demonstrated that while the levels of HCV Ab in GCF were slightly elevated (by 2%) above those in the saliva, the difference was not statistically significant (*P* > .05). As mentioned by the authors [13, 14], this result may be partially due to the presence of PCR inhibitors in saliva. In studies carried out by Maticic et al. [13] and Suzuki et al. [14] which have qualitatively identified HCV in GCF and saliva, HCV-RNA were detected 59.35% and 85.31%, respectively. Their results were higher than ours. These discrepancies may be due to collecting specimens from GCF and saliva. We tried to rule out the possible effect of small amount of bleeding as a source of HCV Ab by using Orthotolidine method [24] but Suzuki et al. cannot rule out the possible effect of small amount of bleeding as a source of HCV-RNA [24]. Furthermore, this difference in findings may originate from the use of volume versus concentration in measurements.

There is a continual transudation of serum into the oral cavity through the junction of the gingival margin with the tooth surface [15]. HCV has been widely detected in blood mononuclear cells (BMCs) in patients with chronic HCV infection, and the differences in quasispecies identification within serum and BMC suggest that viral replication occurs within BMC [10–12, 38]. HCV infected BMC might allow HCV to infiltrate the GCF and saliva. Generally, periodontal inflammation increases the excretion of BMC-rich GCF. However, no correlation was found between HCV-RNA in saliva oral health and viral load [27, 36]. Therefore, HCV has been identified in the mucosal tissue as well as in the salivary glands of anti-HCV positive patients with oral lichen planus [8].

Thus, it is likely that several possible sources discussed above are involved in HCV penetration into the saliva and GCF. Epidemiological studies, however, suggest that the infective capacity of HCV viral particles in saliva is low, but it has not been possible to determine their infective potential. Moreover, HCV-specific receptors have not been defined on oral epithelial cells nor has the role of host defence mechanims been determined. New experimental animal models and the recently described infectious HCV pseudoparticles, capable of simulating HCV replication in vitro, could be useful in establishing any role of saliva in the transmission of HCV infection [39]. However, a further investigation is needed to understand the source of the HCV-RNA in saliva and whether it represents an increased risk of transmission.

## 5. Conclusion

It is evident that patients with chronic renal disease will comprise on enlarging proportion of the dental patients population in the future [40]. Our study showed that HCV Ab is present in saliva and GCF in patients with hemodialysis. Hence, practitioners need to be aware of the precautions necessary in treating patients with hemodialysis.

## Figures and Tables

**Table 1:** Crosstabulation of HCV antibodies Immunoreactivity in Gingival Crevicular fluid and Saliva, Kappa = 0.426; *p *< .001.

		Gingival Crevicular fluid							
		Positive		Gray Zone		Negative		Total	
		*n*	%	*n*	%	*n*	%	*n*	%
Saliva	Positive	2	5.1			3	7.7	5	12.8
	Gray Zone			3	7.7			3	7.7
	Negative	4	10.3	1	2.6	26	66.7	31	79.5
	Total	6	15.4	4	10.3	29	74.4	39	100

## References

[1] Nakayama E, Akiba T, Marumo F, Sato C (2000). Prognosis of anti-hepatitis C virus antibody-positive patients on regular hemodialysis therapy. *Journal of the American Society of Nephrology*.

[2] Moroni GA, Cori P, Marelli F (1991). Indirect evidence for transfusion role in in conditioning hepatitis C-virus prevalence among hemodialysis patients. *Nephron*.

[3] Fabris P, Infantolino D, Biasin MR (1999). High prevalence of HCV-RNA in the saliva cell fraction of patients with chronic hepatitis C but no evidence of HCV transmission among sexual partners. *Infection*.

[4] Wang JT, Wang TH, Sheu JC, Lin JT, Chen DS (1992). Hepatitis C virus RNA in saliva of patients with post-transfusion hepatitis and low efficiency of transfussion among spouses. *Journal of Medical Virology*.

[5] Liou T-C, Chang T-T, Young K-C, Lin X-Z, Lin C-Y, Wu H-L (1992). Detection of HCV RNA in saliva, urine, seminal fluid, and ascites. *Journal of Medical Virology*.

[6] Fried MW, Shindo M, Fong T-L, Fox PC, Hoofnagle JH, Di Bisceglie AM (1992). Absence of hepatitis C viral RNA from saliva and semen of patients with chronic hepatitis C. *Gastroenterology*.

[7] Hermida M, Ferreiro MC, Barral S, Laredo R, Castro A, Diz Dios P (2002). Detection of HCV RNA in saliva of patients with hepatitis C virus infection by using a highly sensitive test. *Journal of Virological Methods*.

[8] Takamatsu K, Koyanagi Y, Okita K, Yamamoto N (1990). Hepatitis C virus RNA in saliva. *The Lancet*.

[9] Couzigou P, Richard L, Dumas F, Schouler L, Fleury H (1993). Detection of HCV-RNA in saliva of patients with chronic hepatitis C. *Gut*.

[10] Wang J-T, Sheu J-C, Lin J-T, Wang T-H, Chen DS (1992). Detection of replicative form of hepatitis C virus RNA in peripheral blood mononuclear cells. *Journal of Infectious Diseases*.

[11] Young K-C, Chang T-T, Liou T-C, Wu H-L (1993). Detection of hepatitis C virus RNA in peripheral blood mononuclear cells and in saliva. *Journal of Medical Virology*.

[12] Takamatsu K, Okayasu I, Koynagi Y, Yamamoto N (1992). Hepatitis C virus propagates in saliva glands. *Journal of Infectious Diseases*.

[13] Matičič M, Poljak M, Kramar B (2001). Detection of hepatitis C virus RNA from gingival crevicular fluid and its relation to virus presence in saliva. *Journal of Periodontology*.

[14] Suzuki T, Omata K, Satoh T (2005). Quantitative detection of hepatitis C virus (HCV) RNA in saliva and gingival crevicular fluid of HCV-infected patients. *Journal of Clinical Microbiology*.

[15] Cimasoni G (1973). *The Crevicular Fluid*.

[16] Yaari A, Tovbin D, Zlotnick M (2006). Detection of HCV salivary antibodies by a simple and rapid test. *Journal of Virological Methods*.

[17] Kuo G, Choo Q-L, Alter HJ (1989). An assay for circulating antibodies to a major etiologic virus of human non-A, non-B hepatitis. *Science*.

[18] Allwright S, Bradley F, Long J, Barry J, Thornton L, Parry JV (2000). Prevalence of antibodies to hepatitis B, hepatitis C, and HIV and risk factors in Irish prisoners: results of a national cross sectional survey. *British Medical Journal*.

[19] Gonzales V, Martro E, Folch C (2008). Detection of hepatitis C virus antibodies in oral fluid specimens for prevalence studies. *European Journal of Clinical Microbiology and Infectious Diseases*.

[20] Üstündağ Y, Hızıl N, Boyacıoğlu S, Akalın E (1997). Detection of hepatitis GB virus-C and HCV genomes in the saliva of patients undergoing maintenance 
haemodialysis. *Nephrology Dialysis Transplantation*.

[21] Bayraktar G, Kazancioğlu R, Bozfakioğlu S, Ecder T, Yildiz A, Ark E (2002). Stimulated salivary flow rate in chronic hemodialysis patients. *Nephron*.

[22] Postorino M, Catalano C, Martorano C (2003). Salivary and lacrimal secretion is reduced in patients with ESRD. *American Journal of Kidney Diseases*.

[23] Judd A, Parry J, Hickman M (2003). Evaluation of a modified commercial assay in detecting antibody to hepatitis C virus in oral fluids and dried blood spots. *Journal of Medical Virology*.

[24] Cox M (1991). A study of the sensitivity and specificity of four presumptive tests for blood. *Journal of Forensic Sciences*.

[25] Hmaied F, Mamou MB, Saune-Sandres K (2006). Hepatitis C virus infection among dialysis patients in Tunisia: incidence and molecular evidence for nosocomial transmission. *Journal of Medical Virology*.

[26] Allander T, Gruber A, Naghavi M (1995). Frequent patient-to-patient transmission of hepatitis C virus in a haematology ward. *The Lancet*.

[27] Chen M, Yun Z-B, Sallberg M (1995). Detection of hepatitis C virus RNA in the cell fraction of saliva before and after oral surgery. *Journal of Medical Virology*.

[28] Coates EA, Brennan D, Logan RM (2000). Hepatitis C infection and associated oral health problems. *Australian Dental Journal*.

[29] De Cock L, Hutse V, Verhaegen E, Quoilin S, Vandenberghe H, Vranckx R (2004). Detection of HCV antibodies in oral fluid. *Journal of Virological Methods*.

[30] Lins L, Almeida H, Vitvisk L, Carmo T, Paraná R, Reis MG (2005). Detection of hepatitis C virus RNA in saliva is not related to oral health status or viral load. *Journal of Medical Virology*.

[31] Dusheiko GM, Smith M, Scheuer PJ (1990). Hepatitis C virus transmitted by human bite. *The Lancet*.

[32] Abe K, Inchauspe G (1991). Transmission of hepatitis C by saliva. *The Lancet*.

[33] Figueiredo JFC, Borges AS, Martinez R (1994). Transmission of hepatitis C virus but not human immunodeficiency virus type 1 by a human bite. *Clinical Infectious Diseases*.

[34] Hsu HH, Wright TL, Lubo D (1991). Failure to detect hepatitis C virus genome in human secretions with the polymerase chain rection. *Hepatology*.

[35] Rey D, Fritsch S, Schmitt C, Meyer P, Lang JM, Stoll-Keller F (2001). Quantitation of hepatitis C virus RNA in saliva and serum of patients coinfected with HCV and human immunodeficiency virus. *Journal of Medical Virology*.

[36] Parra B, Slots J (1996). Detection of human viruses in periodontal pockets using polymerase chain reaction. *Oral Microbiology and Immunology*.

[37] Chebbi F, Poveda J-D, Suzuki T (1997). Search for infectious HIV in gingival crevicular fluid and saliva of advanced AIDS patients with severe periodontitis. *AIDS*.

[38] Qian C, Camps J, Maluenda MD, Civeira MP, Prieto J (1992). Replication of hepatitis C virus in peripheral blood mononuclear cells. Effect of alpha-interferon therapy. *Journal of Hepatology*.

[39] Bartosch B, Dubuisson J, Cosset F-L (2003). Infectious hepatitis C virus pseudo-particles containing functional E_1_-E_2_ envelope protein complexes. *Journal of Experimental Medicine*.

[40] Craig RG (2008). Interactions between chronic renal disease and periodontal disease. *Oral Diseases*.

